# Determination and characterisation of the surface charge properties of the bacteriophage M13 to assist bio-nanoengineering†

**DOI:** 10.1039/d0ra04086j

**Published:** 2020-07-03

**Authors:** Paolo Passaretti, Yiwei Sun, Timothy R. Dafforn, Pola Goldberg Oppenheimer

**Affiliations:** Institute of Cancer and Genomic Science, University of Birmingham Birmingham B15 2TT UK p.passaretti@bham.ac.uk; School of Engineering and Materials Science, Queen Mary University of London London E1 4NS UK; School of Biosciences, University of Birmingham Birmingham B15 2TT UK; School of Chemical Engineering, University of Birmingham Birmingham B15 2TT UK P.GoldbergOppenheimer@bham.ac.uk

## Abstract

To truly understand the mechanisms behind the supramolecular self-assembly of nanocomponents, the characterisation of their surface properties is crucial. M13 emerged as a practical nanocomponent for bio-nanoassemblies of functional materials and devices, and its popularity is increasing as time goes by. The investigation performed in this study provides important information about the surface charge and the surface area of M13 determined through the comparison of structural data and the measurement of *ζ*-potential at pH ranging between 2 and 11. The developed methodologies along with the experimental findings can be subsequently exploited as a novel and convenient prediction tool of the total charge of modified versions of M13. This, in turn, will facilitate the design of the self-assembly strategies which would combine the virus building block with other micro and nano components *via* intermolecular interactions.

## Abstract 2

Characterisation of the external surface of bacteriophage M13 using PVIII protein structural data and measuring *ζ*-potential of the entire virus.

## Graphical abstract

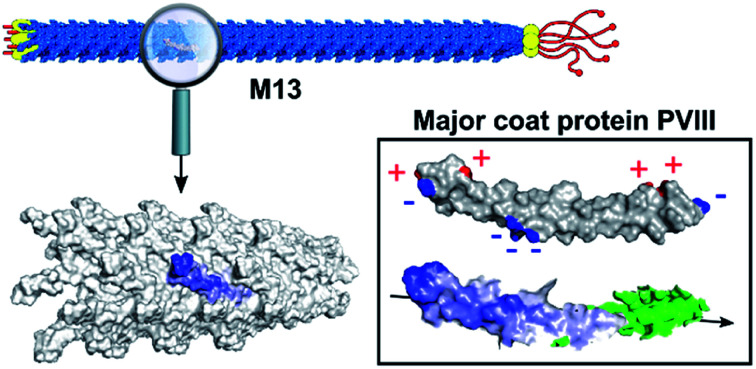

## Introduction

M13 is a filamentous bacteriophage that infects *Escherichia coli* (*E. coli*) strains showing F-*pilus*.^1^ First isolated by P. Hofschneider in 1963, it was subsequently characterised and widely employed in various scientific fields including, for instance, molecular biology and nanotechnology.^2–5^ This virus is very well known for the development of the phage display,^6^ which allows fused peptides and proteins on the virion capsid to be exposed. Moreover, M13 is a versatile tool for the fabrication of nanostructured materials due to its readiness for functionalisation and compatibility with other molecules.^7–9^

M13 is comprised of a circular single-stranded DNA (ssDNA) molecule surrounded by a protein capsid, which measures ∼1 μm in length and ∼6 nm in width. Although its genome encodes for eleven proteins (PI–XI), its protein capsid is only comprised of five of them. Approximately 2700 copies of the major coat protein PVIII, constitute the majority of the phage capsid. About five copies of each of the minor coat proteins PIII and PVI constitute one end of the virion, while five copies of each of the PIX and PVII constitute the other.^2,10^

Supramolecular chemistry exploits every kind of interactions, ranging from hydrogen to covalent bonds to assemble into complex hierarchical structures. Although the covalent bonds are stronger than other types of interactions, given the extremely small dimensions of nanomaterials, even the weaker intermolecular forces (IMFs) can significantly affect their self-assembly processes. Therefore, it is fundamental to explore and characterise all the possible interactions of this nanocomponent.

To understand the structure of the PVIII protein of M13, it is important to understand which chemical bonds can be employed for the display of functional groups as well as to predict possible interactions through IMFs for the design of novel self-assembled systems. Despite numerous studies describing the ionic properties of other similar filamentous phages, there is currently no available information specifically focussed on the M13 bacteriophage.^11–13^ Zimmermann *et al.* characterised the surface charge of the two analogous filamentous bacteriophages *i.e.*, Pf1 and *fd via* polyelectrolyte titration across the pH range between 3 and 9. The study confirmed that the DNA charge of both phages is neutralised by the interaction with the capsid proteins. Moreover, the isoelectric point (IEP) of both viruses was evaluated at 4.0 and 4.2 as well as their surface charge density of 0.46 eq. nm^2^ and 0.48 eq. nm^2^ for Pf1 and *fd*, respectively.^13^ Both *fd* and Pf1 are filamentous bacteriophages, belonging to class I and II, respectively. The enterobacteria phage *fd* has a genome sequence homology of 96.99% compared to M13 (class I) and therefore, it is considered an “almost identical” analogue. On the other hand, Pf1 has a genome sequence homology of 50.11% compared to the M13, its genome is slightly bigger (7349 bp) and the whole phage is more than twice as long. However, their coat proteins have similar secondary and tertiary structures (ESI Table S1†).^14,15^

Our study is specifically focussed on the M13. This virus was systematically characterised, combining both structural information and the measurement of *ζ*-potential to establish which PVIII amino acids contribute to its total charge and subsequently, which chemical groups could influence the assembly of M13-based supramolecular structures. Our study thus lays the platform for expanding approaches to modify M13 and characterise more in detail its interactions with other components. The methodology described was designed specifically for the M13 wild type, however, it can further be adapted to predict the total charge of modified versions of M13 displaying added charged chemical groups or peptides on its external surface as well as to study the surface charge of other viruses.

Chemical and genetic modifications of M13 are largely exploited in the field of bio-nanoengineering for the fabrication of nanostructured materials.^7–9^ In particular, genetic modification allows the display of specific peptides that can bind a variety of molecules for the fabrication of biosensors^16–18^ and electronic materials^19^ as well as target cancerous cells,^20–24^ bind factors to direct cell proliferation and differentiation for tissue regeneration.^25^ The presence of ionisable groups in these foreign peptides can drastically change the chemical characteristics of the virus, especially when fused to the major coat protein PVIII. Therefore, it is important to predict how these modifications can affect the IEP and other surface properties of these modified viruses.

To predict these, we have analysed the PVIII protein using a 3D model on Protein Data Bank (PDB) (ID: 2MJZ) and all the potentially charged amino acids in the range of pH between 2 and 11 were identified in the protein sequence. Their charge and the absolute p*K*_a_ (p*K*_a_1__) values were collected from the data available in the literature.^26^ Subsequently, both the level of exposure to the solvent and an alternative p*K*_a_ (p*K*_a_2__), which considers the chemical surrounding of these charged residues were calculated using pyMOL and PROPKA.^27–29^ These parameters were employed to both predict the surface charge of M13 across the whole range of pH and to estimate its IEP. These theoretical models were compared to the experimental *ζ*-potential of M13 measured between pH 2 and 11. Furthermore, based on the same protein structure described above, the surface area and molecular weight of M13 were calculated. Finally, our study has also yielded an R script, which can calculate the IEP of modified versions of the M13 *via* providing the specific p*K*_a_ values along with the level of exposure of the ionisable groups present on the major coat protein.

Considering the increasing amount of publication related to the fabrication of self-assembled nanostructures and devices involving the M13 bacteriophage, our work will be beneficial to predict the potential interaction of bio-nanocomponents for a broad range of physical science, chemistry, nanotechnology, biotechnology and medical communities.

## Results and discussion

### Characterisation of the PVIII protein

**Fig. 1:**
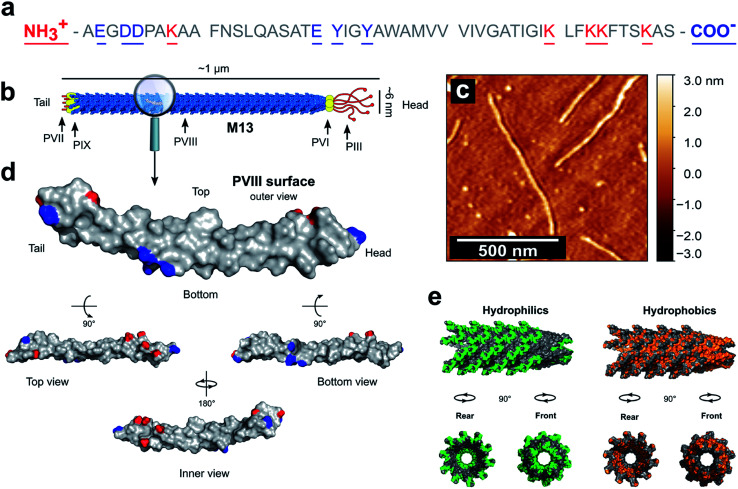
PVIII sequence and characteristics. (a) The sequence of the PVIII protein and (b) the schematic representation of M13. (c) AFM image of individual M13 phages on a silicon substrate. (d) An individual PVIII structure with the ionizable species highlighted in blue and red corresponding to the negatively and positively charged residues respectively (PDB 2MJZ).^30^ The non-ionizable species are coloured in grey. (e) In green is shown the hydrophilic surface corresponding to the amino acids R, K, H, E, D, N, Q, T, S and C, while the hydrophobic surface (orange) corresponds to the residues A, G, V, I, L, F and M.

PVIII is the main component of M13 and is of a major presence on the surface of the virion. The total mass of M13 was calculated to be 16 430 kDa including the mass of the ssDNA and both minor and major coat proteins (ESI Table S2†). Moreover, the PVIII corresponds approximately to 98% of the mass of the capsid and 86% of the mass of the virion, in agreement with the measured values shown in the literature (ESI Table S3†).^30,31^ This suggests that the PVIII provides the greatest contribution to the total surface and charge of the M13 particle. Therefore, due to their marginal influence on the total charge of M13 and to simplify our model, a detailed description of the minor coat proteins was not included. Determining the p*K*_a_ values of the amino acids in PVIII is therefore crucial for calculating both the total charge of M13 at pH values ranging from 2 to 11 and its isoelectric point (IEP). The mature PVIII protein is an α-helix that forms a helical capsid wrapping the viral single-stranded genomic DNA (ssDNA).^13^ It is made of fifty amino acids. Eleven of these, and the amino- and carboxyl-terminal groups can sensibly contribute to its total charge (Fig. 1a).^30^

The carboxyl group at the C-terminus as well as at the terminal K40, K43, K44 and K48 residues are exposed in the internal cavity of the capsid, stabilising the entire bacteriophage structure whilst interacting with the negative charges distributed along the sugar-phosphate backbone of the ssDNA.^13^ Therefore, these residues are completely embedded in the viral structure and thus, do not make a significant contribution to the total charge on the external surface of the M13.

Residues Y21 and Y24, have high p*K*_a_ values (Table 1), making their contribution to the total charge at pH > 10 quite considerable. However, they are predominantly screened by other residues and contribute marginally the overall surface charge.

**Table 1:** PVIII protein p*K*_a_ values

PVIII amino acid	Amino acid portion	Charge	p*K*_a_1__	p*K*_a_2__	Exposure
A1	N-terminus	+	9.69	8.62	100%
E2	Variable group	−	4.25	3.45	100%
D4	Variable group	−	3.65	3.11	100%
D5	Variable group	−	3.65	4.02	58%
K8	Variable group	+	10.53	11.56	100%
E20	Variable group	−	4.25	5.21	75%
Y21	Variable group	−	10.07	14.74	30%
Y24	Variable group	−	10.07	13.14	77%
K40	Variable group	+	10.53	9.67	63%^a^
K43	Variable group	+	10.53	11.17	61%^a^
K44	Variable group	+	10.53	9.91	48%^a^
K48	Variable group	+	10.53	8.16	30%^a^
S50	C-terminus	−	2.21	2.18	47%^a^

a The percentage of exposure of these residues refers to the area facing the internal cavity of the virion.

Therefore, we hypothesised that the amino group at the N-terminus, together with the residues E2, D4, D5, K8 and E20 are the main contributors to the overall charge of the M13, due to their chemical properties and external location on the virion surface (Fig. 1d).

### The surface area of M13

**Fig. 2:**
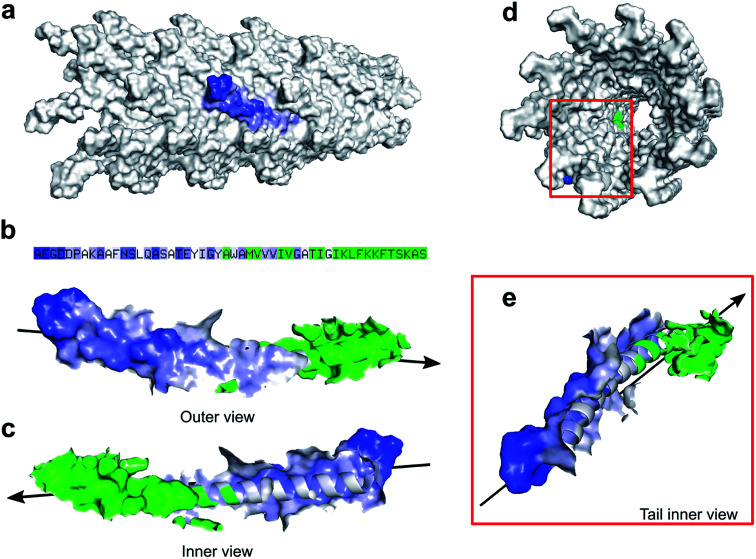
M13 surface area. (a) A portion of the M13 capsid (grey) with an individual PVIII coloured in blue at different intensities. The darker blue regions indicate higher accessibility to the solvent of that specific area. (b) The sequence in colour code and the PVIII outer view, showing the exposed surfaces and (c) its inner view from the internal cavity of the virion. The residues exposed in the internal cavity and buried under other PVIIIs are coloured in green. The arrow indicates the extremity of the virus where the PIII and PVI are located. This end is also considered the ‘head’ of the virus. (d) Back view of the M13 capsid shows the exposed surfaces of a PVIII highlighted in blue and green. (e) Magnified PVIII as described above.

Zimmermann *et al.* calculated the surface area of M13 to be 18 300 nm^2^ considering the virus as a cylinder-like structure measuring 1 μm in length and 6 nm in diameter.^13^ However, pyMOL enables a more accurate estimation of the surface area allowing us to introduce an alternative value which to our knowledge, is not available in the literature. Therefore, all the residues exposed on the external surface of an individual PVIII assembled in the M13 capsid were identified and the others, facing the internal cavity, were excluded from the selection. Subsequently, the surface of the externally exposed amino acids was calculated and coloured in blue, having the intensity directly proportional to the level of exposure (Fig. 2a).

The hidden residues and those facing the internal cavity were coloured in green and excluded from the calculation of the external surface. The solvent-accessible surface area of the PVIII not assembled in the capsid was found to be 4658 ± 58 Å^2^ (Fig. S4a†), while the sum of the external and internal (facing the inner cavity) surfaces of a PVIII assembled in the capsid was found to be 1801 ± 20 Å^2^ (Fig. 2b). The surface of the residues exposed on the external surface was measured as 1349 ± 17 Å^2^ (blue gradient residues). The latter value, multiplied by the number of PVIII copies (2700), equals to 36 433 nm^2^ (ESI Table S4 and Fig. S4†). This value corresponds to the total surface of M13 excluding the contribution of minor coat proteins and the different structural characteristics present at the two ends of the virion. Although the calculated surface area has not been determined experimentally, we propose this as an alternative value, obtained *via* summing the solvent-accessible surface area of the external portion of 2700 PVIII proteins, in addition to the one proposed by Zimmerman *et al.*, which corresponds to the area of a cylinder having the dimensions of M13.

### The surface charge of M13

**Fig. 3:**
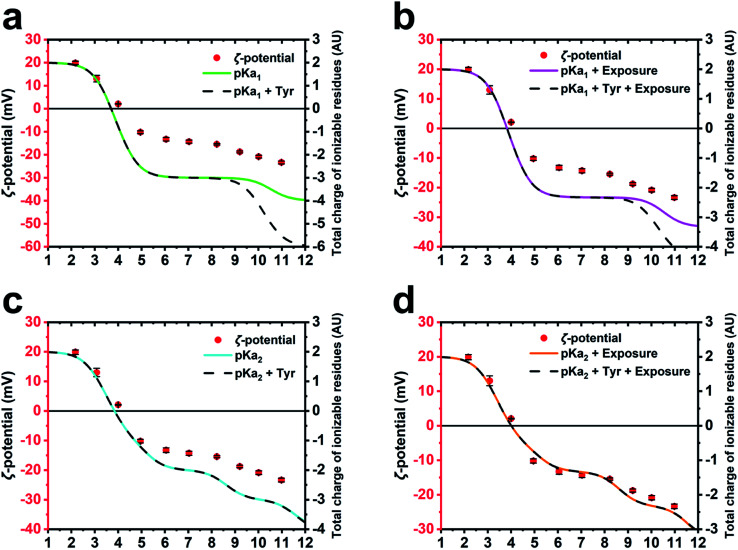
*ζ*-Potential and predicted curves of M13 charge. Mathematical models compared with the experimentally measured *ζ*-potential of the M13 (red dots). (a) The models are based on p*K*_a_1__ (green) and the same including the contribution of the internal tyrosines (Y21 and Y24) (dash line). (b) The models employing p*K*_a_1__ (magenta) and also the contribution of the internal tyrosines (dash line), including residues exposure in both cases. (c) The models employing p*K*_a_2__ (cyan) and including the contribution of the internal tyrosines (dash line). (d) The models employing p*K*_a_2__ (orange) and the contribution of the internal tyrosines (dash line), including residues exposure in both cases.

To further corroborate the hypothesis that the charge of M13 is predominantly given by a certain group of PVIII amino acids and to calculate the IEP of the bacteriophage, several models of charge potential curve models were compared to the *ζ*-potential of M13 measured at pH 2–11 (Fig. 3). Each model was made summing different combination of the charge of Y24, Y21, E20, K8, D5, D4, E2 and the amino group of A1 calculated using (i) their absolute p*K*_a_ found in the literature (p*K*_a_1__),^26^ (ii) their p*K*_a_ influenced by the chemical surrounding in the M13 capsid (p*K*_a_2__) and their level of exposure, both calculated using pyMOL/PROPKA.

Table 1 shows the kind of charge, the solvent exposure and the p*K*_a_ values of the ionizable amino acids on the external surface of M13. The p*K*_a1_ values refer to the variable portion of these residues.^26^ However, it is important to consider that the p*K*_a_ of the amino acids are influenced by their chemical surroundings, especially if they are part of a polypeptide like in this case. Therefore, an alternative p*K*_a_ (p*K*_a_2__) was calculated for each amino acid using the bioinformatic software pyMOL implemented with PROPKA.^27–29^ The latter is a popular bioinformatic tool which can predict the p*K*_a_ of the amino acid variable portion, considering the influence given by the presence of the near chemical groups that can alter their absolute p*K*_a_.^27–29^ The amino acids in Table 1 are divided into three groups based on their position, including the external surface of the phage, the middle portion of the PVIII protein and the internal cavity of the viral capsid (starred residues).^10^ Moreover, the calculation performed by PROPKA provides information about the percentage of exposure to the solvent for the amino acids under consideration.^27–29^ Therefore, the charge of each residue was calculated considering two important parameters: the area of the charged amino acids exposed on the surface of M13, expressed in percentage and the influence of the other surrounding chemical groups on the reactivity of those.

Based on the p*K*_a_ and exposure values obtained with PROPKA, mathematical models of the virus charge were calculated employing the Henderson–Hasselbalch equation to predict the degree of ionization of each ionizable amino acid.^32^ According to these calculations, 99% of the species were protonated or deprotonated at 2 pH units either side of the pH corresponding to their p*K*_a_2__.^32^

The total charge of M13 was calculated by different mathematical models adopting different parameters of the PVIII protein residues such as p*K*_a_1__, p*K*_a_2__, the contribution of the Y21 and Y24 (Tyr) and the exposure level of the charged residues (ESI Table S5–S8†). The different predictions were compared with the experimentally measured *ζ*-potential of M13 (Fig. 3a–d). The different models were compared to each other and to the *ζ*-potential, to check whether the model values were diverging from the data obtained experimentally.

While the M13 charge models calculated using only p*K*_a_1__ and the same combined with Tyr were found to overlap for the pH values ranging from 1 to 8.5, the model influenced by Tyr was found to exhibit greater negativity at pH ranging from 8.5 to 12 (Fig. 3a). This is attributed to the high p*K*_a_ values of the Tyr. The comparison with the *ζ*-potential trend shows similarities only at the pH values < 9 (Fig. 3a).

The M13 charge was calculated with the model using p*K*_a_1__ and the same combined with Tyr, but in this case taking into account their percentage of exposure to the solvent (Fig. 3b). These models show a very similar trend to the previous ones (Fig. 3a), although they are more similar to the curve obtained from the measurement of the *ζ*-potential, highlighting the importance of considering the exposure of the charged residues in estimating the total charge of the M13.

Subsequently, the M13 charge was re-calculated by similar models using the p*K*_a_2__ instead of p*K*_a_1__ (Fig. 3c and d). Although the model excluding the residual exposure level has a more similar trend to the measured *ζ*-potential, compared to the models in Fig. 3a and b, the models calculated using p*K*_a_2__, Tyr and including the exposure level of the charged residues (Fig. 3d), shows the best fit to the measured *ζ*-potential values. This suggests that the amino group at the N-terminus, the E2, D4, D5, K8 and E20 residues make the greatest contribution to the formation of the surface charge of M13. This is further corroborated by the abundance of the PVIII protein (∼2700 copies) compared to the total of the minor coat proteins (∼20) along with the inability of the ssDNA molecule to contribute due to its negative charge, and enclosure by the PVIII. Furthermore, it is important to note that while considering the residues contribution, we must take into account the variation of the p*K*_a_, which is on itself influenced by the chemical environment as well as the degree of exposure. However, the tyrosines Y21 and Y24 do not appear to be crucial in the total charge of M13 given their extremely high p*K*_a_ values.

Our study established the IEP of M13 at 4.05 ± 0.065. This was obtained averaging the *ζ*-potential and the theoretical curves values (ESI Fig. S2†). Similar values were previously measured for F-specific filamentous viruses (Ff) and the *fd* bacteriophage, which have an identical, mature PVIII protein, apart for the substitution N35D.^12,13^ Finally, the charged external surface is calculated to cover an area of approximately 14 283 nm^2^ corresponding to the 39% of the total surface of M13.

### Applications and future perspectives

This study provides important information on the surface area of M13 and its surface charge, which were subsequently used for the determination of its IEP. To produce novel nano-assemblies, it is necessary to be able to control the M13 *via* suitable parameters of pH, temperature and compatibility to the solvent in which it is dispersed. Also, other components that have their own chemical and physical properties are often used for the nano-assembly processes and therefore, to combine M13 with such additional components, the reaction conditions must be specific and finely tuned. The IEP of M13, in particular, determines its stability in solution as well as provides indications regarding the net charge given by the ionisable groups exposed on its surface. The accurate determination of this value was, for instance, crucial in our previous work, where M13 was self-assembled with graphene oxide (GO) to form a porous aerogel based on weak intermolecular interactions.^33^ The R script (ESI Code S1†) enables the calculation of the M13's IEP as well as the determination of how this value would change when different ionizable amino acids are added to the PVIII.^34^ Furthermore, this script could be useful for calculating the IEP of a modified version of M13 carrying additional peptides on the major coat protein. This, in turn, would enable planning which residues are most suitable for the specific purposes in a time-efficient manner. Collectively, our method can enable the design of alternative versions of the M13 both, chemically modified and genetically engineered, to be employed in the nano-assemblies based both on intermolecular interactions and covalent bonds.^35–38^

## Conclusions

The self-assembly of M13-based nanostructures and material is a growing trend, especially aimed at the production of biosensors, scaffolds, batteries, supercapacitors and other electronic components.

The extremely small size of M13 and other nanocomponents employed to manufacture such devices, allow the electrostatic forces and other IMFs to significantly affect their assembly. Therefore, we have studied the chemical properties of the external surface of the M13 bacteriophage and developed a predictive model of its surface charge. This can be used *via* the R script detailed in the ESI† to predict the charge of alternative versions of M13 obtained through genetic and chemical modifications.

We experimentally found that the IEP of the virion is 4.05 ± 0.065 and its charge is predominantly due to the charged residues of the major coat protein PVIII. The amino acids A1, E2, D4, D5, K8 and E20 are the most exposed on the external surface of M13 and provide the major contribution to the overall charge. Their p*K*_a_ is influenced by the surrounding chemical environment and the level of exposure to the solvent. Therefore, it is crucial to consider these conditions in the calculation of the total charge. Residues Y21 and Y24 should be also considered for inclusion in the calculations however, these are protonated at pH ranging from 1 to 12 and therefore, do not contribute to the total charge. We have also calculated the surface area and the molecular weight of the virus to be 36 433 nm^2^ and 16 430 kDa, respectively. Although the values were not obtained experimentally, they are of significant importance for the design of nano-assemblies, where the surface area of M13 was typically obtained *via* just an approximation to a surface of a cylinder of dimensions similar to the virus.

The developed method and the R script available in the ESI† are a facile approach to predict and compare the total charge of genetically modified versions of the M13, which is of special interest and importance for the future fabrication of novel M13-based self-assembled nanostructures and nanomaterials. Finally, this versatile methodology can be further adapted to other viruses and proteins further employable for the design, development and exploitation of novel bio-nanoassemblies.

## Materials and methods

### M13 propagation and purification

M13 bacteriophage (M13KE) was purchased from New England Biolabs® as double-stranded DNA (dsDNA) and transferred into One Shot™ TOP10F′ Chemically Competent *E. coli* (Thermo Fisher Scientific®) through heat shock. M13 was self-propagated in batch cultures using the aforementioned *E. coli* strain. For the batch propagation were employed, Nutrient Broth No. 2 (Thermo Fisher Scientific® Oxoid) dissolved with PBS at pH 7.3 (Thermo Fisher Scientific® Oxoid) in presence of tetracycline of 5 μg mL^−1^ and incubating M13 and *E. coli* at 37 °C, 150 rpm for 24 h. After the propagation, the culture was centrifuged two times at 8000 rpm (Beckman Coulter® JA 10, RCF = 11 295*g*) and the pellet containing *E. coli* (brown pellet) was discarded. The supernatant containing M13 was subsequently incubated with PEG 6000 (Sigma-Aldrich®) and stirred for 90 min at 4 °C. The supernatant/PEG solution was centrifuged at 10 000 rpm (Beckman Coulter JA 10®, RCF = 17 649*g*) and the supernatant discarded. The obtained white pellet mainly consisting of M13, was resuspended in DIW. The newly purified stock was subject to isoelectric precipitation at pH 4 using HCl 5 M. Therefore, after flocculation, the virus was pelleted at 15 000 rpm for 1 min (SciSpin Mini Microfuge RCF = 15 100*g*), the supernatant discarded, and the pellet resuspended in DIW. Further centrifugation at 15 000 rpm for 1 min helped to pellet any insoluble fractions leftover and collect the supernatant containing M13. The concentration was finally determined *via* UV-Vis spectrophotometry (Thermo Fisher Scientific® Evolution 600) measuring the absorbance at 269 nm (*ε* = 3.84 ± 0.06 cm^2^ mg^−1^).^39^

### Atomic force microscopy (AFM)

AFM (BRUKER Innova®) images were acquired on a p-type silicon wafer (Sigma-Aldrich®) in dry conditions. A drop of 20 μL containing M13 of 25 μg mL^−1^ in DIW was drop-cast onto a ∼25 mm^2^ silicon wafer and let dry at room temperature. The images were subsequently acquired in tapping mode with BRUKER® RTESPA-300 probe (*T*: 3.4 μm; *L*: 125 μm; *W*: 40 μm; *f*_0_: 300 kHz; *k*: 40 N m^−1^).

### Describe *ζ*-potential measurement

M13 of 1 mg mL^−1^ was dispersed in several tubes with 10 mM NaCl with a pH ranging from pH 2 to 11 and analysed with the particle analyser (Malvern Zetasizer Nano ZS®). The analysis shows that M13 charge ranges from −23.36 ± 0.65 to 19.88 ± 0.74 at pH 10.98 and 2.17 respectively (Table 2).

**Table 2:** M13 *ζ*-potential at different pH values

pH	*ζ*-Potential (mV)	SD
2.17	19.88	±0.74
3.08	13.02	±1.43
4.00	2.07	±0.08
4.95	−10.22	±0.61
6.05	−13.26	±0.77
7.03	−14.32	±0.68
8.22	−15.46	±0.33
9.20	−18.78	±0.44
10.01	−20.86	±0.62
10.98	−23.36	±0.65

## Conflicts of interest

There are no conflicts to declare.

## Supplementary Material

RA-010-D0RA04086J-s001
