# Supplementary material for: Determination and characterisation of the surface charge properties of the bacteriophage M13 to assist bio-nanoengineering
Source: RSC Adv. 2020 Jul 3;10(42):25385–92. doi: 10.1039/d0ra04086j (PMC9055230; doi:10.1039/d0ra04086j)
Supplement: RA-010-D0RA04086J-s001 [file RA-010-D0RA04086J-s001.pdf]

Electronic Supplementary Material (ESI) for RSC Advances.
This journal is © The Royal Society of Chemistry 2020

Supplementary Information

Determination and Characterisation of the Surface Charge Properties of the Bacteriophage M13 to Assist Bio-nanoengineering

Paolo Passaretti,[a] Yiwei Sun,[b] Timothy R. Dafforn[c] and Pola Goldberg Oppenheimer[d]*

[a] Institute of Cancer and Genomic Science, University of Birmingham, Birmingham, B15 2TT, UK
[b] School of Engineering and Materials Science, Queen Mary University of London, London, E1 4NS, UK
[c] School of Biosciences, University of Birmingham, Birmingham, B15 2TT, UK
[d] School of Chemical Engineering, University of Birmingham, Birmingham, B15 2TT, UK

Table of Contents

1. **Table S1** 2
2. **Table S2** 3
3. **Table S3** 3
4. **Table S4** 4
5. **Figure S1** 5
6. **Table S5** 6
7. **Table S6** 8
8. **Table S7** 10
9. **Table S8** 12
10. **Code S1** 14
11. **Code S2** 15
12. **Figure S2** 16
13. **References** 17

Table S1. Comparison between of M13, *fd* and Pf1

	M13	Ref.	*fd*	Ref.	Pf1	Ref.
**Ff filamentous phage class**	I		I		II	
**Genome ID**	NC_003287.2		J02451.1		NC_001331.1	
**Genome type**	Circular ssDNA		Circular ssDNA		Circular ssDNA	
**Genome length (bp)**	6407		6408		7349	
**Percent Identity Matrix – Clustal 2.1**	100%		96.99%		50.11%	
**Virion length (nm)**	850-900	1,2	760-870	2	~ 2000	3
**Virion diameter (nm)**	~ 6.6	1	~ 5.6	2	~ 7	3
**Number of major coat proteins per phage**	2700	4	2700	4	7600	4
**Coat proteins**	PIII, PVI, PVII, PVIII and PIX	2	III, VI, VII, VIII and IX	5	p3, p6, p7, p8 and p9	6
**Molecular weight (Da)**	16.8×10^6	7	16.4×10^6	8	37.5×10^6	4

Further information on the differences between M13, *fd* and Pf1 can be found in the literature.[3,4,9–12]

Table S2. Molecular weight of M13 with calculated values

	Amino acids (aa)	Base pairs (bp)	Number of copies	MW (Da)	MW_{tot} (Da)
**PIII**	406	n/a	5	42 579.09	212 895.45
**PVI**	112		5	12 350.09	61 750.45
**PVII**	33		5	3 602.24	18 011.20
**PIX**	32		5	3 653.29	18 266.45
**PVIII**	50		2700	5 238.04	14 142 708.00
**ssDNA**	n/a	6407	1	1 976 583.36	n/a
**Mass of protein capsid:** 14 453 631.55 Da					
**Mass of protein capsid and nucleic acid:** 16 430 214.91 Da					

The molecular weight (MW) of proteins shown in **Table S2** were calculated using ExPASy Compute pI/Mw tool, available at the following link https://web.expasy.org/compute_pi/.[13–15] The MW of the ssDNA (M13 genome sequence available here https://www.ebi.ac.uk/ena/data/view/V00604&display=fasta) was calculated using DNA calculator (nucleic acid parameters: DNA; single strand; circular), available at the following link http://www.molbiotools.com/dnacalculator.html.

Table S3. Molecular weight of M13 with measured values

	Amino acids (aa)	Base pairs (bp)	Number of copies	MW (Da)	MW_{tot} (Da)	References
**PIII**	406	n/a	5	42 675	213 375	[16]
**PVI**	112		5	12 264	61 320	
**PVII**	33		5	3 587	17 935	
**PIX**	32		5	3 654	18 270	
**PVIII**	50		2700	5 234	14 131 800	
**ssDNA**	n/a	6407	1	2×10^6	n/a	[2,17]
**Mass of protein capsid:** 14 442 700 Da						
**Mass of protein capsid and nucleic acid:** 16 442 700 Da						

The total mass calculated is in agreement with the value proposed by Beck *et al.* ($16.8 \times 10^6 \pm 0.8$ Da)[7] and with the values obtained *via* combining the data in **Table S3**.

Table S4. M13 surface areas

	PDB ID: 2MJZ	T	U	V	W	X	Y	Z	a	b	c	d	e	f	g	h	Average (Å²)	Subunits sum (Å²)	M13 areas (Å²)	M13 areas (nm²)	M13 areas (µm²)
Molecular surface	Free	5381	5379	5380	5378	5381	5380	5379	5574	5379	5380	5380	5380	5382	5377	5379	5393 ± 48				
	Assembled	5287	5285	5287	5285	5287	5284	5282	5283	5282	5283	5286	5286	5288	5283	5286	5285 ± 2	79,273	14,269,205	142692	0.143
	Core																	81,193	14,614,726	146,147	0.146
	Asbl_ext	3051	3051	3051	3050	3052	3051	3052	3050	3051	3051	3054	3054	3054	3053	3054	3052 ± 1	45,777	8,239,940	82,399	0.082
	Core_ext																	46,029	8,285,259	82,853	0.083
	Asbl_int	2232	2231	2232	2231	2231	2229	2227	2229	2227	2228	2229	2228	2230	2226	2228	2229 ± 2	33,438	6,018,801	60,188	0.060
	Core_int																	33,053	5,949,506	59,495	0.059
Solvent accessible surface	Free	4636	4639	4636	4636	4634	4635	4634	4807	4807	4634	4636	4634	4634	4636	4636	4658 ± 58				
	Assembled	1820	1820	1817	1817	1818	1772	1774	1775	1774	1771	1811	1809	1810	1811	1810	1801 ± 20	27,009	4,861,577	48,616	0.049
	Core																	27,021	4,863,846	48,638	0.049
	Asbl_ext	1337	1339	1335	1336	1337	1336	1337	1338	1337	1334	1374	1372	1373	1374	1373	1349 ± 17	20,233	3,641,851	36,419	0.036
	Core_ext																	20,241	3,643,327	36,433	0.036
	Asbl_int	483	482	482	482	481	436	437	438	437	436	437	437	437	437	437	452 ± 21	6779	1,220,141	12,201	0.012
	Core_int																	6776	1,219,697	12,197	0.012

**Free**	The area of each PVIII protein taken individually, not assembled to form the capsid
**Assembled**	The area of each PVIII protein assembled to form the capsid, excluding their unexposed portions due to the interactions with the surrounding PVIIIs
**Core**	The area of the capsid portion composed of the 15 chains named T, U, V, W, X, Y, Z, a, b, c, d, e, f, g and h from the PDB file 2MJZ, excluding their unexposed portions
**Asbl_ext**	The area of each assembled PVIII protein excluding the area of the residues in green (inner portion). This corresponds to the contribution of a single PVIII to the M13 external total surface.<br>AEGDDPAKAAFNSLQASATEYIGY AWAMVVVIVGATIGIKLFKKFTSKAS
**Core_ext**	The area of the core excluding the area of the residues in green (inner portion). This corresponds to the contribution of 15 PVIIIs to the M13 external total surface area.<br>AEGDDPAKAAFNSLQASATEYIGY AWAMVVVIVGATIGIKLFKKFTSKAS
**Asbl_int**	The area of each assembled PVIII protein excluding the area of the residues in blue (external portion). This corresponds to the contribution of a single PVIII to the M13 internal total surface.<br>AEGDDPAKAAFNSLQASATEYIGY AWAMVVVIVGATIGIKLFKKFTSKAS
**Core_int**	The area of the core excluding the area of the residues in blue (external portion). This corresponds to the contribution of 15 PVIIIs to the M13 internal total surface area.<br>AEGDDPAKAAFNSLQASATEYIGY AWAMVVVIVGATIGIKLFKKFTSKAS

The surface area in **Table S4** were calculated with pyMOL selecting individual PVIII chains (free and assembled) and the central 15 chains all together (core). The values were calculated using the molecular and solvent accessible surface parameters, respectively. Note, that the values of subunits sum of Assembled, Asbl_ext and Asbl_int correspond to the sum of the chains between T to h, while Core, Core_ext and Core_int correspond to the values of the 15 chains taken all together. The M13 areas were calculated as: (Subunits sum/15)*2700.

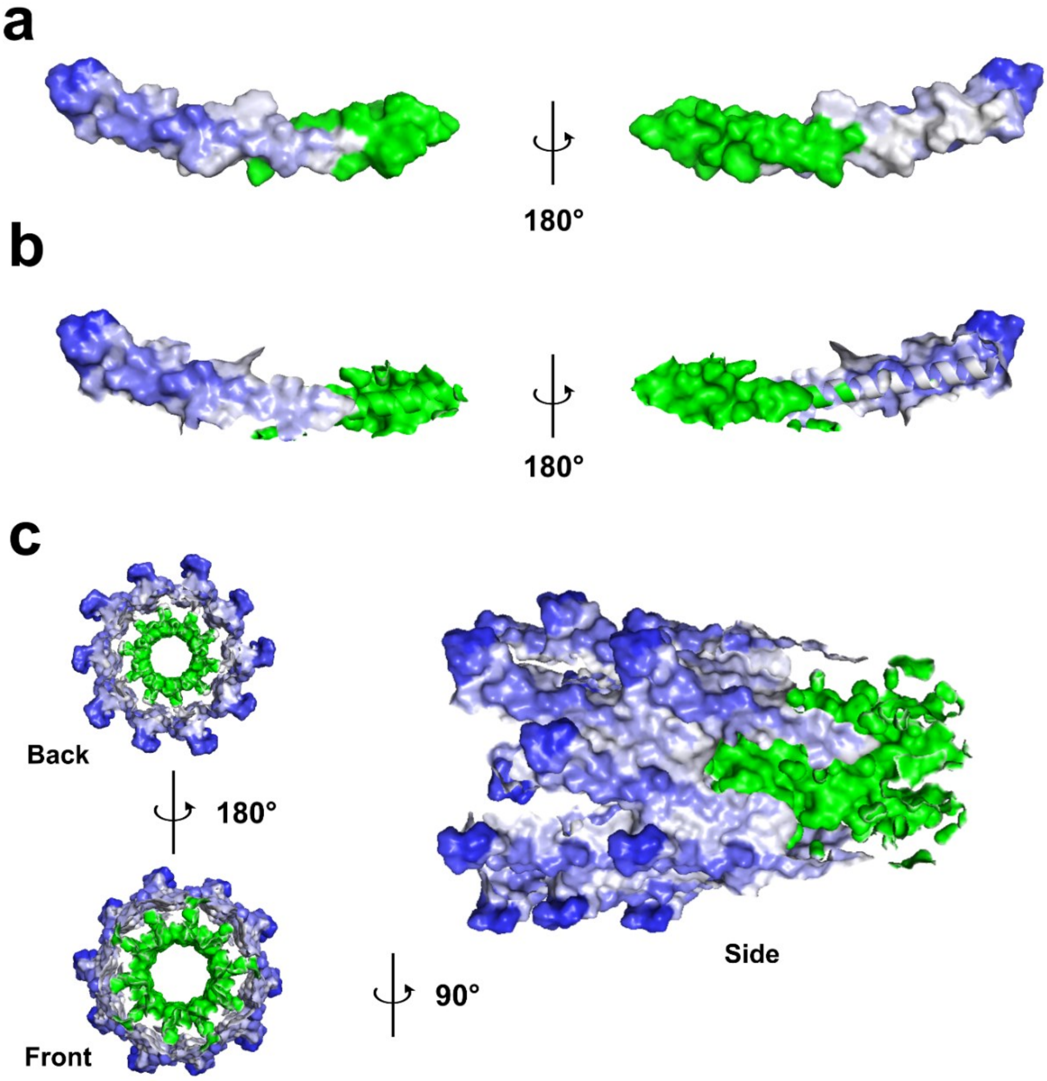

Figure S1. M13 surface areas

The surface of PVIII according to Table S4: **(a)** free, **(b)** assembled and **(c)** core.

Table S5. pKa_1 models

Title	Equation	Y axes: charge – X axes: pH
**A1**	(1 / (10^(ph - 3.69) + 1)) * (1)	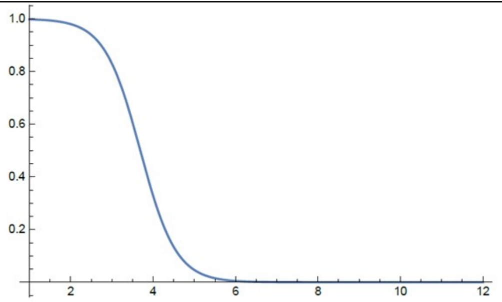
**A2**	(1 - 1 / (10^(ph - 4.25) + 1)) * (-1)	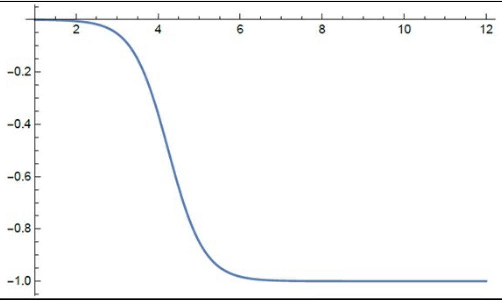
**A3**	(1 - 1 / (10^(ph - 3.65) + 1)) * (-1)	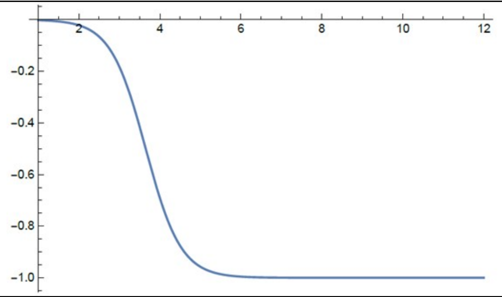
**A4**	(1 - 1 / (10^(ph - 3.65) + 1)) * (-1)	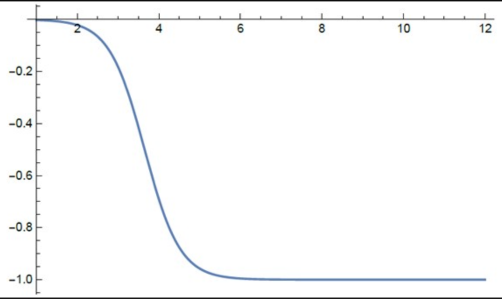
**A5**	(1 / (10^(ph - 10.53) + 1)) * (1)	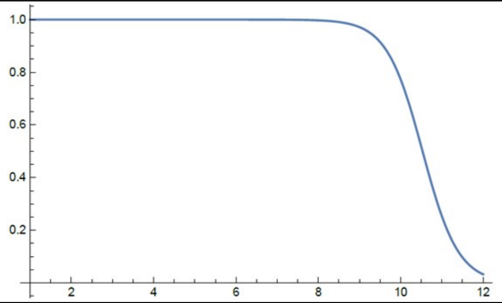

**A6**	(1 - 1 / (10^(ph - 4.25) + 1)) * (-1)	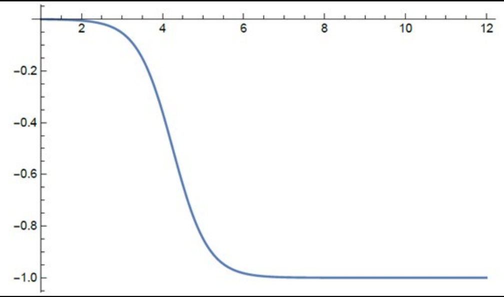
**A7**	(1 - 1 / (10^(ph - 10.07) + 1)) * (-1)	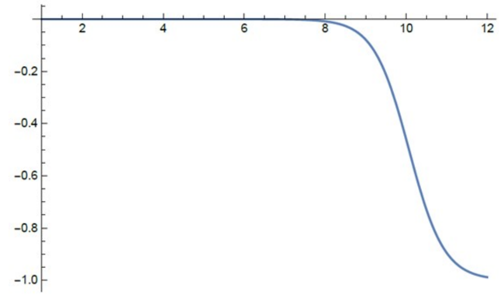
**A8**	(1 - 1 / (10^(ph - 10.07) + 1)) * (-1)	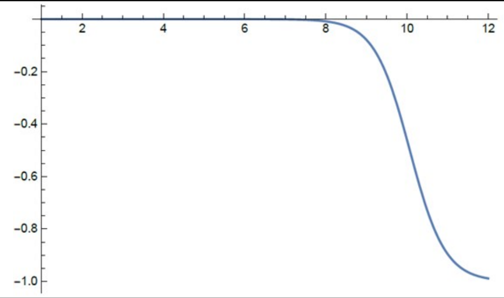
	A_{tot} = A1 + A2 + A3 + A4 + A5 + A6 + A7 + A8	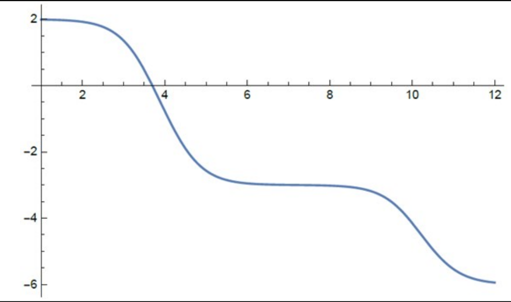
	A_{tot} = A1 + A2 + A3 + A4 + A5 + A6	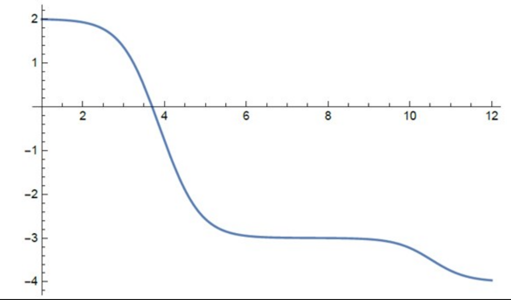

Table S6. pKa$_1$ + exposure models

Title	Equation	Y axes: charge – X axes: pH
**B1**	(1 / (10^(ph - 3.69) + 1)) * (1)	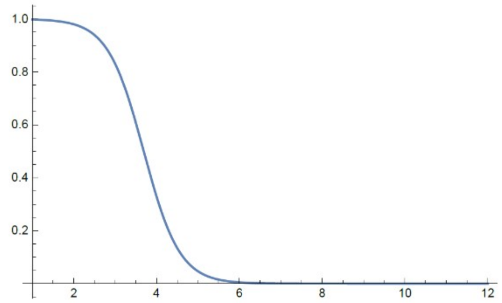
**B2**	(1 - 1 / (10^(ph - 4.25) + 1)) * (-1)	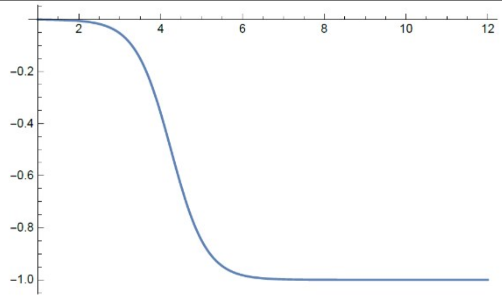
**B3**	(1 - 1 / (10^(ph - 3.65) + 1)) * (-1)	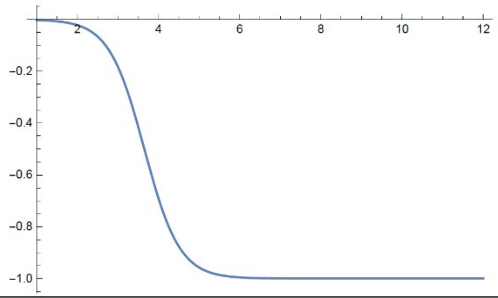
**B4**	(1 - 1 / (10^(ph - 3.65) + 1)) * (-0.58)	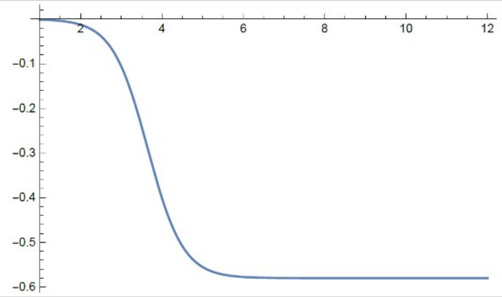
**B5**	(1 / (10^(ph - 10.53) + 1)) * (1)	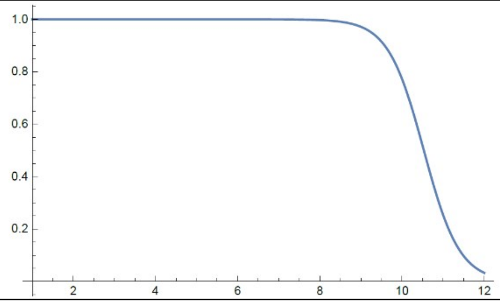

**B6**	(1 - 1 / (10^(ph - 4.25) + 1)) * (-0.75)	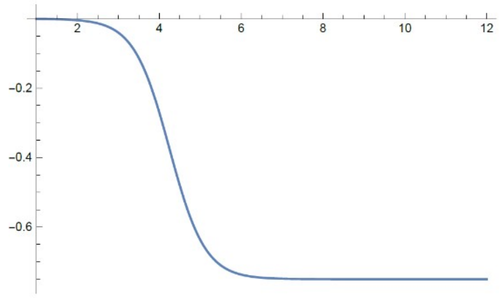
**B7**	(1 - 1 / (10^(ph - 10.07) + 1)) * (-0.30)	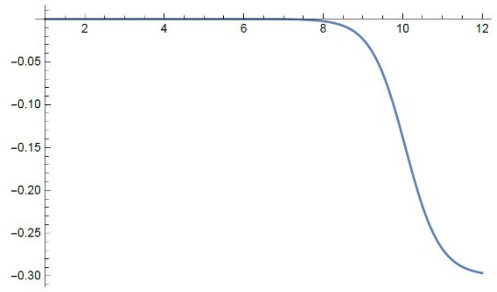
**B8**	(1 - 1 / (10^(ph - 10.07) + 1)) * (-0.77)	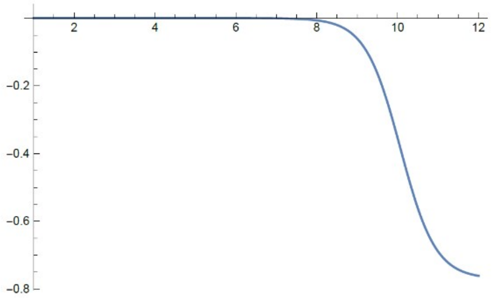
	B_{tot} = B1 + B2 + B3 + B4 + B5 + B6 + B7 + B8	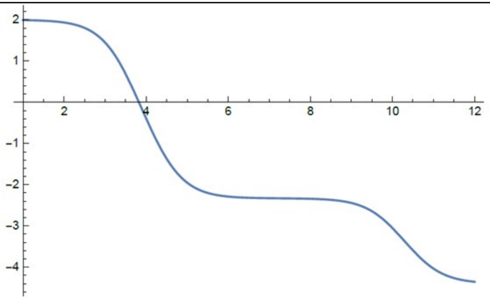
	B_{tot} = B1 + B2 + B3 + B4 + B5 + B6	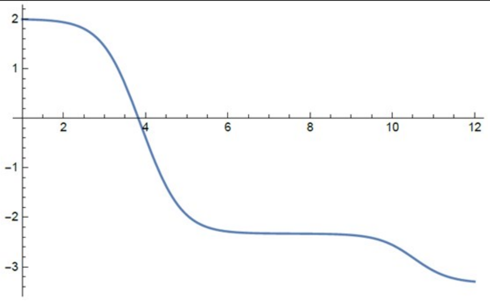

Table S7. pKa_2 models

Title	Equation	Y axes: charge – X axes: pH
**C1**	(1 / (10^(ph - 8.62) + 1)) * (1)	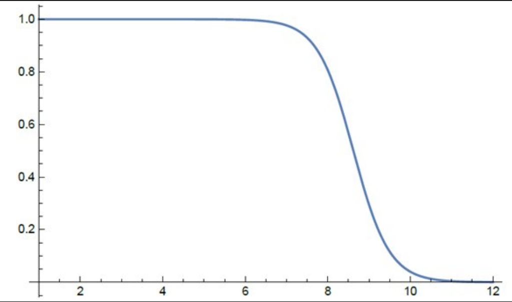
**C2**	(1 - 1 / (10^(ph - 3.45) + 1)) * (-1)	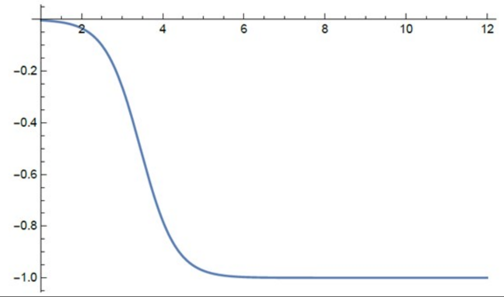
**C3**	(1 - 1 / (10^(ph - 3.11) + 1)) * (-1)	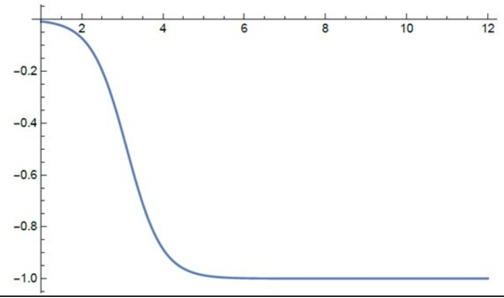
**C4**	(1 - 1 / (10^(ph - 4.02) + 1)) * (-1)	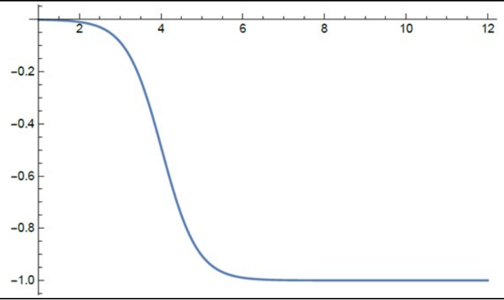
**C5**	(1 / (10^(ph - 11.56) + 1)) * (1)	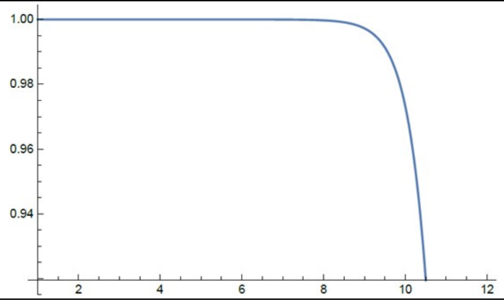

**C6**	(1 - 1 / (10^(ph - 5.21) + 1)) * (-1)	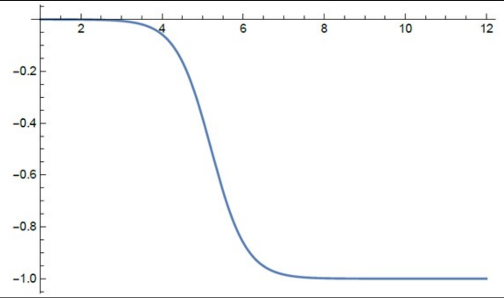
**C7**	(1 - 1 / (10^(ph - 14.74) + 1)) * (-1)	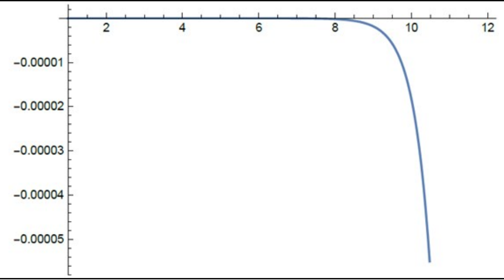
**C8**	(1 - 1 / (10^(ph - 13.14) + 1)) * (-1)	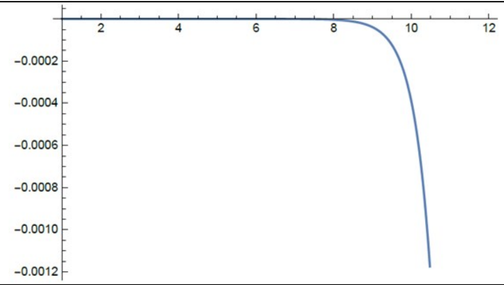
	C_{tot} = C1 + C2 + C3 + C4 + C5 + C6 + C7 + C8	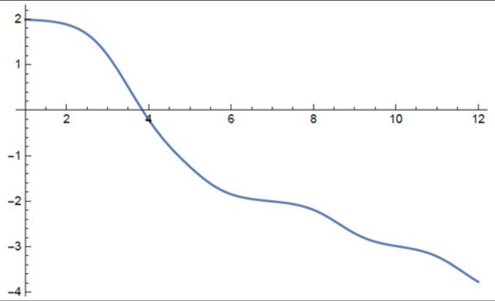
	C_{tot} = C1 + C2 + C3 + C4 + C5 + C6	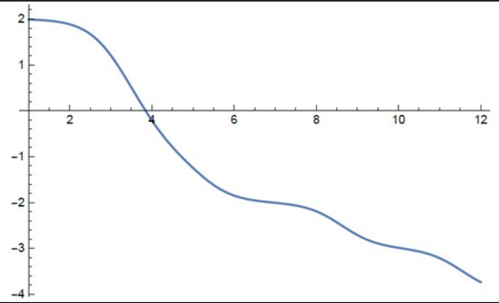

Table S8. pKa$_2$ + exposure models

Title	Equation	Y axes: charge – X axes: pH
**D1**	(1 / (10^(ph - 8.62) + 1)) * (1)	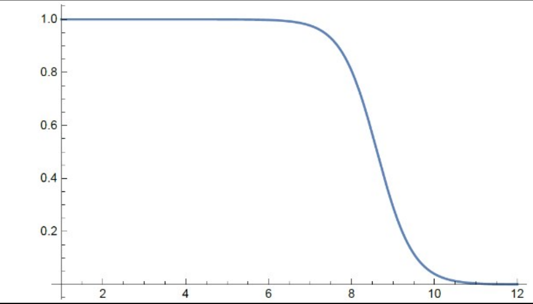
**D2**	(1 - 1 / (10^(ph - 3.45) + 1)) * (-1)	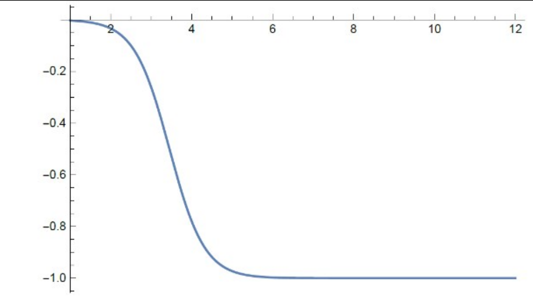
**D3**	(1 - 1 / (10^(ph - 3.11) + 1)) * (-1)	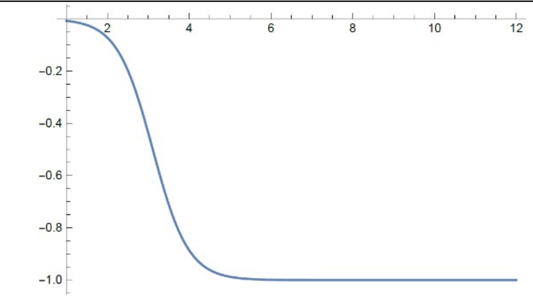
**D4**	(1 - 1 / (10^(ph - 4.02) + 1)) * (-0.58)	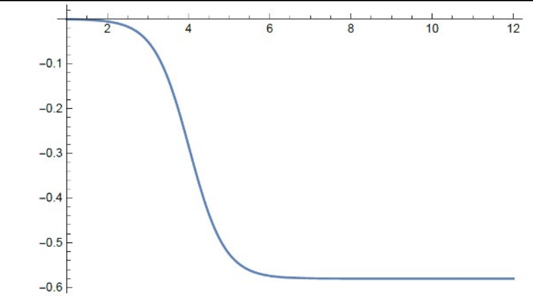
**D5**	(1 / (10^(ph - 11.56) + 1)) * (1)	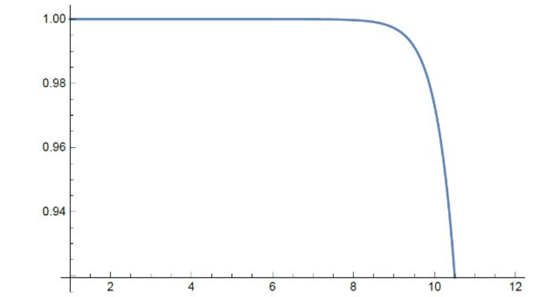

**D6**	(1 - 1 / (10^(ph - 5.21) + 1)) * (-0.75)	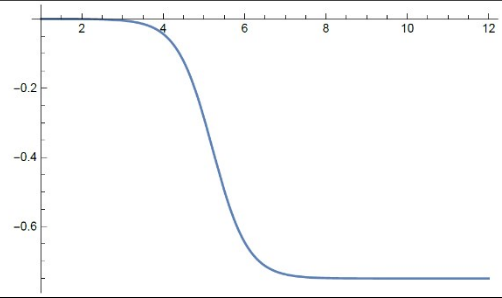
**D7**	(1 - 1 / (10^(ph - 14.74) + 1)) * (-0.30)	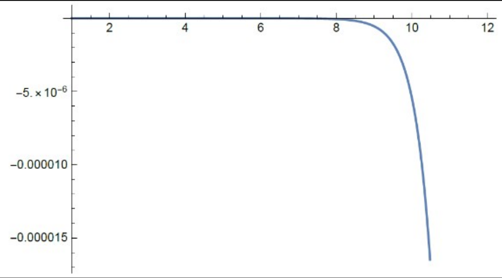
**D8**	(1 - 1 / (10^(ph - 13.14) + 1)) * (-0.77)	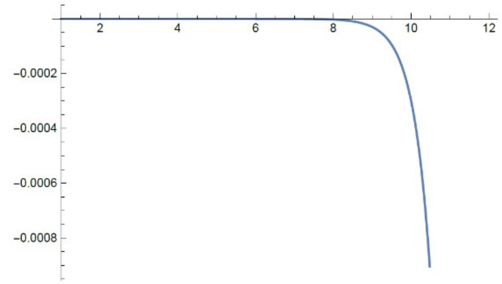
	D_{tot} = D1 + D2 + D3 + D4 + D5 + D6 + D7 + D8	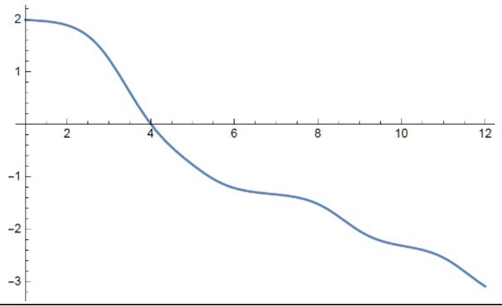
	D_{tot} = D1 + D2 + D3 + D4 + D5 + D6	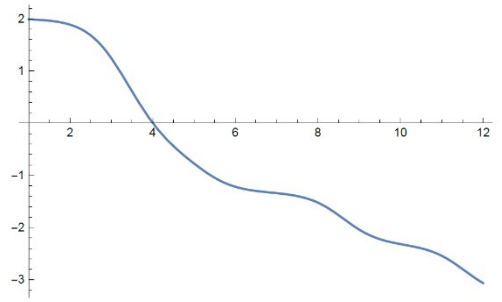

Following the code to calculate the charge of the PVIII protein based on the selected amino acids (A1, E2, D4, D5, K8, E20, Y21, Y24). The following code can be used in R to calculate the IEP of modified versions of the PVIII changing some some parameter accordingly **(Code S1)**. More information on how to use it are included in the code as well as two examples are listed below **(Code S2 and S3)**.

Code S1. IEP calculator for PVIII protein and other modified versions

```
# Activate the necessary libraries
library(ggplot2)

# Building the X axes which is the pH scale
pH <- seq(1, 12, by=0.001)

# The amino acid listed below are the residues contributing to the total charge
of the M13 wild type
# Adjust the value row:9 column:12 accordingly with the number of residues of
your modified M13
num <- c(1:8)

# Add in brackets the amino acid name and position of your modified M13
name <- c("A1", "E2", "D4", "D5", "K8", "E20", "Y21", "Y24")

# Add the pKa of the corresponding amino acid
pka <- c(8.64, 3.45, 3.11, 4.02, 11.56, 5.21, 14.74, 13.14)

# Add 1 or 0 if the residue is positively or negatively charged
char <- c(1, 0, 0, 0, 1, 0, 0, 0)

# Add the level of exposure to the solvent of the corresponding residue
exp <- c(1, 1, 1, 0.58, 1, 0.75, 0.3, 0.77)

# Create a data frame with all data
PVIII <- data.frame(num, name, pka, char, exp)
PVIII

for(i in num){
if(PVIII$char[i] == 1){
  assign(sprintf("amino.acid_%d", i), ((1/(10^(pH - PVIII$pka[i]) + 1)) *
(PVIII$exp[i])), envir = .GlobalEnv)
  }
  else {
  assign(sprintf("amino.acid_%d", i), ((1-1/(10^(pH - PVIII$pka[i]) + 1)) * (-
PVIII$exp[i])), envir = .GlobalEnv)
  }
 rm(i)
}

# Once obtained the vectors named "amino.acis_n", if more than 8, add them to the
vector tot (Eg: amino.acid_9 + amino.acid_10)
tot <- amino.acid_1 + amino.acid_2 + amino.acid_3 + amino.acid_4 +
       amino.acid_5 + amino.acid_6 + amino.acid_7 + amino.acid_8

# IEP is the isoelectric point generated from the modified PVIII
IEP <- pH[which.min(abs(tot-0))]
# IEP2 is the closest value to zero of the generated charge values
IEP2 <- tot[which.min(abs(tot-0))]

result <- data.frame(pH, tot, IEP2)
result

plotIEP <- ggplot(result, aes(x=pH, y=tot, lable=pH)) +
           geom_point(size=0.5, shape=1, color="red") +
           geom_text(aes(label=ifelse(tot==IEP2,as.character(pH),'')),hjust=-
0.5,vjust=-1.5) +
           xlab("pH") +
           ylab("Number of charges") +
           theme_light()
plotIEP <- plotIEP + geom_hline(yintercept=0, linetype="solid", color = "black",
size=0.8)
plotIEP <- plotIEP + geom_vline(xintercept=IEP, linetype="solid", color =
"black", size=0.8)
plotIEP
```

Code S2. IEP calculator used to calculate a modified version of the PVIII

```
# Example: PVIII protein with 3 aspartic and 1 glutamic acids at the N-terminus
in position 2, 3, 4 and 5
pH <- seq(1, 12, by=0.001)
num <- c(1:12)
name <- c("A1", "D2x", "D3x", "D4x", "E5x" "E2", "D4", "D5", "K8", "E20", "Y21",
"Y24")
pka <- c(8.64, 3.11, 3.11, 3.11, 3.45, 3.45, 3.11, 4.02, 11.56, 5.21, 14.74,
13.14)
char <- c(1, 1, 1, 1, 0, 0, 0, 0, 1, 0, 0, 0)
exp <- c(1, 1, 1, 1, 1, 1, 1, 0.58, 1, 0.75, 0.3, 0.77)
PVIII <- data.frame(num, name, pka, char, exp)
PVIII
for(i in num){
  if(PVIII$char[i] == 1){
    assign(sprintf("amino.acid_%d", i), ((1/(10^(pH - PVIII$pka[i]) + 1)) *
(PVIII$exp[i])), envir = .GlobalEnv)
  }
  else {
    assign(sprintf("amino.acid_%d", i), ((1-1/(10^(pH - PVIII$pka[i]) + 1)) * (-
PVIII$exp[i])), envir = .GlobalEnv)
  }
  rm(i)
}
tot <- amino.acid_1 + amino.acid_2 + amino.acid_3 + amino.acid_4 + amino.acid_5 +
amino.acid_6 + amino.acid_7 + amino.acid_8 +
  amino.acid_9 + amino.acid_10 + amino.acid_11 + amino.acid_12
IEP <- pH[which.min(abs(tot-0))]
IEP2 <- tot[which.min(abs(tot-0))]
result <- data.frame(pH, tot, IEP2)
result
plotIEP <- ggplot(result, aes(x=pH, y=tot, lable=pH)) +
  geom_point(size=0.5, shape=1, color="red") +
  geom_text(aes(label=ifelse(tot==IEP2,as.character(pH),'')),hjust=-0.5,vjust=-
1.5) +
  xlab("pH") +
  ylab("Number of charges") +
  theme_light()
plotIEP <- plotIEP + geom_hline(yintercept=0, linetype="solid", color = "black",
size=0.8)
plotIEP <- plotIEP + geom_vline(xintercept=IEP, linetype="solid", color =
"black", size=0.8)
plotIEP
```

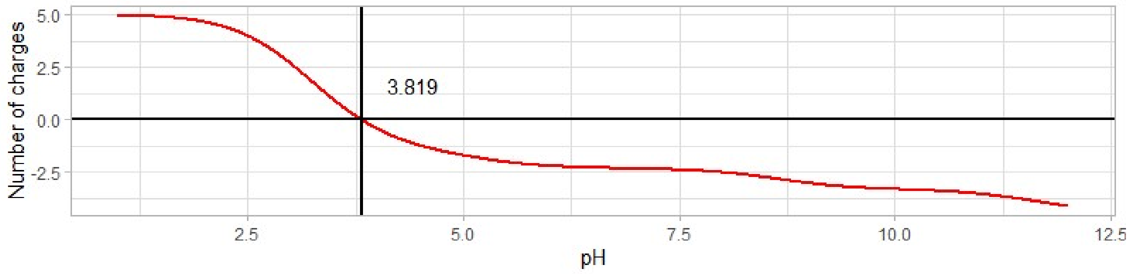

a

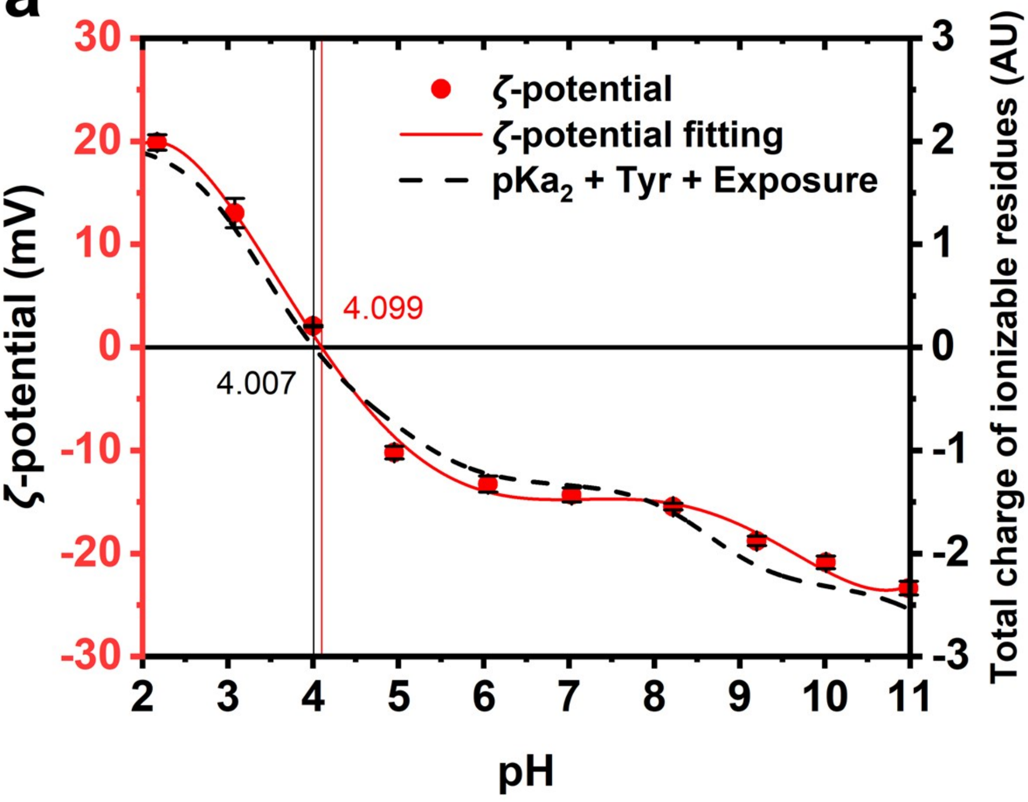

b

Equation	y = Intercept + B1*x^1 + B2*x^2 + B3*x^3 + B4*x^4 + B5*x^5	
Plot	z-potential	error
Weight	No Weighting	
Intercept	-75.82086 ± 30.16783	-4.10503 ± 12.07138
B1	116.81266 ± 30.39556	5.47588 ± 12.16251
B2	-49.08253 ± 11.21275	-2.15651 ± 4.48668
B3	8.55142 ± 1.91684	0.3817 ± 0.76701
B4	-0.67362 ± 0.15362	-0.03129 ± 0.06147
B5	0.01979 ± 0.00466	9.66421E-4 ± 0.00187
Residual Sum of Squares	5.38041	0.86147
R-Square (COD)	0.99726	0.2131
Adj. R-Square	0.99384	-0.77053

Figure S2. Determination of the IEP of M13

(a) The ζ-potential values were fitted with a polynomial function of order 5 and compared to the model pKa_2 which includes tyrosins and exposure level. The final IEP value was calculated *via* averaging the IEPs fom both curves. **(b)** The parameters of the fitting function.

References

1. Marvin, D. A. & Hoffmann-Berling, H. Physical and Chemical Properties of Two New Small Bacteriophages. *Nat. Publ. Gr.* **4866**, 517–518 (1963).

2. Marvin, D. A. & Hohn, B. Filamentous Bacterial Viruses. *Bacteriol. Rev.* **33**, 172–209 (1969).

3. Wolkers, W. F., Haris, P. I., Pistorius, A. M. A., Chapman, D. & Hemminga, M. A. FT-IR Spectroscopy of the Major Coat Protein of M13 and Pf1 in the Phage and Reconstituted into Phospholipid Systems. *Biochemistry* **34**, 7825–7833 (1995).

4. Torbet, J. & Maret, G. High-field magnetic birefringence study of the structure of rodlike phages Pf1 and fd in solution. *Biopolymers* **20**, 2657–2669 (1981).

5. Marvin, D. A. Filamentous Phage Structure, Infection and Assembly. *Curr. Opin. Struct. Biol.* **8**, 150–158 (1998).

6. Stubbs, G. & Kendall, A. Helical viruses. *Adv. Exp. Med. Biol.* **726**, 631–658 (2012).

7. Beck, K. & Duenk, R. M. Flexibility of Bacteriophage M13: Comparison of Hydrodynamic Measurements with Electron Microscopy. *J. Struct. Biol.* **105**, 22–27 (1990).

8. Forsheit, A. B. & Ray, D. S. Conformations of the Single-Stranded DNA of Bacteriophage M13. *Proc. Natl. Acad. Sci.* **67**, 1534–1541 (1970).

9. Day, L. A. & Wiseman, R. L. A Comparison of DNA Packaging in the Virions of fd , Xf , and Pf1. *Cold Spring Harb. Monogr. Arch.* **8**, 605–625 (1978).

10. Day, L. A., Casadevall, A., Prescott, B. & Thomas, G. J. Raman Spectroscopy of Mercury(II) Binding to Two Filamentous Viruses: Ff (fd, M13, f1) and Pf1. *Biochemistry* **27**, 706–711 (1988).

11. Casadevall, A. & Day, L. A. DNA Packing in the Filamentous Viruses fd, Xf, Pfl and Pf3. *Nucleic Acids Res.* **10**, 2467–2481 (1982).

12. Hill, D. F., Short, N. J., Perham, R. N. & Petersen', G. B. *DNA Sequence of the Filamentous Bacteriophage Pfl. I. Nol. Biol* vol. 218 (1991).

13. Bjellqvist, B. *et al.* The Focusing Positions of Polypeptides in Immobilized pH Gradients can be Predicted from their Amino Acid Sequences. *Electrophoresis* (1993) doi:10.1002/elps.11501401163.

14. Bjellqvist, B., Basse, B., Olsen, E. & Celis, J. E. Reference points for comparisons of two-dimensional maps of proteins from different human cell types defined in a pH scale where isoelectric points correlate with polypeptide compositions. *Electrophoresis* **15**, 529–539 (1994).

15. Gasteiger, E. *et al. The Proteomics Protocols Handbook - Chapter 52: Protein Identification and Analysis Tools on the ExPASy Server*. *The Proteomics Protocols Handbook* (2005). doi:10.1385/1592598900.

16. van Wezenbeek, P. M. G. F. G. F., Hulsebos, T. J. M. M. & Schoenmakers, J. G. G. G. Nucleotide Sequence of the Filamentous Bacteriophage M13 DNA Genome: Comparison with Phage fd. *Gene* **11**, 129–148 (1980).

17. Ray, D. S., Bscheider, H. P. & Hofschneider, P. H. Replication of the Single-Stranded DNA of the Male-Specific Bacteriophage M13. Isolation of Intracellular Forms of Phage-Specific DNA. *J. Mol. Biol.* **21**, 473–483 (1966).
